# Measuring the prevalence and impact of poor menstrual hygiene management: a quantitative survey of schoolgirls in rural Uganda

**DOI:** 10.1136/bmjopen-2016-012596

**Published:** 2016-12-30

**Authors:** Julie Hennegan, Catherine Dolan, Maryalice Wu, Linda Scott, Paul Montgomery

**Affiliations:** 1 Centre for Evidence-Based Intervention, University of Oxford, Oxford, UK; 2 SOAS, University of London, London, UK; 3 Applied Technologies for Learning in the Arts and Sciences, University of Illinois, Urbana-Champaign, Urbana, Illinois, USA; 4 Said Business School, University of Oxford, Oxford UK

**Keywords:** menstrual hygiene management, adolescent girls, education, reproductive heatlh, risk factors, EPIDEMIOLOGY

## Abstract

**Objectives:**

The primary objective was to describe Ugandan schoolgirls’ menstrual hygiene management (MHM) practices and estimate the prevalence of inadequate MHM. Second, to assess the relative contribution of aspects of MHM to health, education and psychosocial outcomes.

**Design:**

Secondary analysis of survey data collected as part of the final follow-up from a controlled trial of reusable sanitary pad and puberty education provision was used to provide a cross-sectional description of girls’ MHM practices and assess relationships with outcomes.

**Setting:**

Rural primary schools in the Kamuli district, Uganda.

**Participants:**

Participants were 205 menstruating schoolgirls (10–19 years) from the eight study sites.

**Primary and secondary outcome measures:**

The prevalence of adequate MHM, consistent with the concept definition, was estimated using dimensions of absorbent used, frequency of absorbent change, washing and drying procedures and privacy. Self-reported health, education (school attendance and engagement) and psychosocial (shame, insecurity, embarrassment) outcomes hypothesised to result from poor MHM were assessed as primary outcomes. Outcomes were measured through English surveys loaded on iPads and administered verbally in the local language.

**Results:**

90.5% (95% CI 85.6% to 93.9%) of girls failed to meet available criteria for adequate MHM, with no significant difference between those using reusable sanitary pads (88.9%, 95% CI 79.0% to 94.4%) and those using existing methods, predominantly cloth (91.5%, 95% CI 85.1% to 95.3%; χ^2^ (1)=0.12, p=0.729). Aspects of MHM predicted some consequences including shame, not standing in class to answer questions and concerns about odour.

**Conclusions:**

This study was the first to assess the prevalence of MHM consistent with the concept definition. Results suggest that when all aspects of menstrual hygiene are considered together, the prevalence is much higher than has previously been reported based on absorbents alone. The work demonstrates an urgent need for improved assessment and reporting of MHM, and for primary research testing the links between menstrual management and health, education and psychosocial consequences.

**Strengths and limitations of this study**

- The study offers a working example of the quantitative assessment of menstrual hygiene management (MHM), using best available evidence to assess each aspect.
- The study provides the first prevalence estimate of adequate MHM consistent with its definition.
- Multivariable comparisons demonstrate the importance of multiple aspects of menstrual hygiene in predicting hypothesised health, education and psychosocial consequences.
- Self-reported menstrual hygiene behaviours, health, education and psychosocial outcomes are vulnerable to biases, particularly social desirability.
- The cross-sectional nature of the study limits causal inference, and the analyses are limited by the lack of ability to adjust for potential sociodemographic confounds, the small sample size and lack of existing literature on which to base power analyses.

## Background

Menstrual hygiene management (MHM) has received increasing attention as a public health issue. After a history of silence, stakeholders from governments to local charities have started to speak about the barrier that the management of menstruation presents to gender equality and the potential for programmes to address the problem. Qualitative research has documented challenges girls face in managing their menses and indicated health, education and psychosocial well-being consequences of poor management.^1–4^ However, quantitative studies of the associations between MHM and health, education and psychosocial outcomes are scarce.^5^ With regard to health, few studies have assessed associations between infection and MHM. In the most comprehensive work to date, a case–control study concluded that there was evidence for an association between reusable pad use and laboratory-confirmed urogenital infection compared with disposable pads, but noted the complex range of MHM practices and the need for more research.^6^ Other physical and health outcomes including irritation, discomfort and urogenital symptoms, even if not confirmed infections, are also important potential consequences of poor MHM which impose on women's ability to participate in society with dignity.^7^ ^8^

School absenteeism and disengagement have been emphasised as important consequences of poor MHM. Quantitative evidence for links between MHM and attendance have been mixed, although studies have almost exclusively looked at absorbent type, rather than all MHM practices discussed below.^9–12^ Studies have focused on attendance,^5^ and additional work is needed to capture the impact of menstruation on concentration and engagement, even if girls are at school.^2^ ^13^ Psychosocial consequences including shame, insecurity, anxiety and fear of stigma are well-documented correlates of menstruation in qualitative studies.^2^ ^4^ Such negative psychosocial outcomes have been hypothesised to arise from poor MHM such as inadequate absorbent use.^3^ They may also result from a lack of knowledge and information about menstruation,^14^ cultural stigma and taboos,^4^ and as a result of transactional or coercive sex undertaken to meet MHM needs.^15^ More quantitative evidence is needed to unpack the roles of these different contributors to well-being, and associations between MHM and well-being outcomes. As argued below, it may be inaccurate to label all of these predictors as MHM.

### MHM: definition, use and measurement

A unified, working definition of MHM was developed by the Joint Monitoring Program of the WHO and UNICEF in 2012, defining MHM as: ‘Women and adolescent girls using a clean menstrual management material to absorb or collect blood that can be changed in privacy as often as necessary for the duration of the menstruation period, using soap and water for washing the body as required, and having access to facilities to dispose of used menstrual management materials’.^16^ This captures aspects of the physical requirements for hygienic, effective management of menstrual bleeding. Use of the term has proliferated; however, operationalisation has been inconsistent. As per the above definition, adequate MHM would require the following:

- clean absorbents
- adequate frequency of absorbent change
- washing the body with soap and water
- adequate disposal
- privacy for managing menstruation.

Studies to date have only reported a few of these aspects (J Hennegan. Menstrual hygiene management and human rights: the case for an evidence-based approach. Under review). Prevalence studies and quantitative assessments of predictors and consequences of poor MHM have focused on the type of absorbent used, the first aspect of MHM.^17^ Some include one or two additional components, such as washing the body or disposal of absorbents.^18^ ^19^ None have included all aspects of MHM or considered a pooled, comprehensive prevalence estimate requiring all conditions to be met. Overextension of the term has also occurred with studies reporting a lack of knowledge about menstruation or cultural taboos as aspects of MHM, rather than contributors to MHM.^12^

One barrier to the full assessment of MHM is the lack of evidence and consensus of what constitutes adequacy in each aspect. In a systematic review of MHM practices and infections, Sumpter and Torondel^20^ found no consistent standard in the application of ‘good’ and ‘bad’ MHM. They found that most studies used only absorbent type to predict consequences and, even with this simplified criterion, did not agree on the adequacy of absorbents such as cloth. Single-use absorbents such as sanitary pads are easily classified as clean; however, reusable absorbents such as cloth or reusable pads (homemade or commercially produced) could be considered hygienic if cleaned appropriately.^20^ MHM literature is yet to provide evidenced guidance for washing and drying absorbents. Washing with soap may be one important criterion. Drying practices are also important, with exposure to UV through sunlight known to have a microbicidal effect.^21^ Leaving fabrics damp has been found to encourage microbial survival, and wearing absorbents wet has frequently been considered unhygienic, with some evidence for greater infection risk and discomfort.^1^ ^6^ ^21^ Similarly, there is little rigorous evidence to guide absorbent change frequency. The *Menstrual Hygiene Matters* report suggested changing absorbents every 2–6 hours dependent on blood flow.^1^ However, there is a lack of evidence on the irritation and infection risk associated with prolonged wear, and women's perceptions of the adequate frequency for changing for comfort and risk of soiling. There is no clear guidance on optimal washing frequency or correct use of soap and water on the body and genitals. Similarly, disposal method adequacy is likely to be contextually dependent and relies on latrine capacity, cultural appropriateness of burning absorbents and other waste disposal methods. Finally, while the concept definition stresses the need to change absorbents in privacy, women and girls need privacy for all aspects of MHM, such as washing the body and absorbents.^7^ Each of these aspects must be considered in trying to estimate the prevalence of MHM, establish relationships between predictors and consequences of poor MHM and evaluate interventions aimed at improving MHM.

Recent calls for action, such as the *MHM in Ten* report,^22^ state the need for a strong quantitative evidence base. This is difficult to achieve without consistent, transparent reports of MHM as defined. Prevalence estimates are needed to assess the distribution of the issue, and to advocate effectively for MHM to be addressed by governments, non-government organisations and other interest groups. Assessment of all aspects of MHM is needed to identify the relevant contributors to the problem, and establish quantitative links between MHM and consequences. For example, a recent systematic review of MHM studies in India reported that in multivariable models, the use of sanitary pads did not predict school attendance.^12^ It is inaccurate to conclude from this finding that MHM does not predict attendance, but only that the type of absorbent used may not. Similarly, trials to date have focused on education and sanitary product provision.^5^ No studies have reported if addressing these contributors to MHM, that is, education informing girls how to manage menstruation hygienically, and the provision of clean absorbents as a resource to improve one aspect of MHM (clean absorbents), actually improves MHM overall. Given the many components of MHM detailed above, it is likely that the provision of menstrual products without attention to other factors is insufficient to improve MHM. This has implications for our expectations and interpretations of intervention outcomes. Trials to date assume that improvements in school attendance or psychosocial outcomes observed are the result of improved MHM, but none have tested this. It may be that other factors such as reductions in menstrual stigma, improved pain management strategies or improved social support among classmates contribute to these improvements. While important aspects of managing menstruation, these factors do not fall under the MHM definition. They risk being minimised if studies focus only on MHM, or being mishandled if inaccurately labelled as MHM, which may further complicate its definition and make measurement more difficult. Thus, there is a need to understand how proposed interventions affect MHM and to establish the role of MHM, in contrast to other aspects of managing menstruation, in hypothesised consequences.

### The present study

This study employs secondary data analysis of the final survey data from the *Menstruation and the Cycle of Poverty* trial undertaken in rural Uganda from January 2012 to December 2014 (Pan African Clinical Trials Registry PACTR201503001044408). The trial methods and outcomes of school attendance and psychosocial well-being are described elsewhere.^23^ Briefly, eight primary schools (including primary school grades 3–7) located in Kamuli district, Uganda, an area characterised by poor performance on education and health indicators, were quasi-randomised to one of four conditions, the provision of: puberty education alone, AFRIpads alone, puberty education and AFRIpads or a no-intervention control. Schools were paired to maximise distance between the four conditions and the risk of contamination. Pairs were allocated sequentially in alphabetical order to conditions. Schools were recruited through the partner NGO and selected to be comparable on characteristics such as size, educational provision and quality.^23^ While water sources were closer to some schools than others, water, sanitation and hygiene (WASH) for MHM was comparable across schools. All girls in the schools in primary school classes 3–5 at baseline were included in the trial, regardless of menstrual status.^23^

Girls in the reusable pad conditions were provided with one pack of AFRIpads and small quantity (one sachet, 45 g) of Omo soap in October 2012, and again in March 2014. Girls were taught about the correct use and cleaning of AFRIpads by local research assistants from the partner NGO on each delivery.^23^ AFRIpads are a washable, reusable cloth sanitary pad produced in Uganda (http://www.afripads.com/). Deluxe packs provided to girls included two soil-resistant plastic-lined ‘base’ pads, three attachable winged liners, three straight liners and two small bags for carrying. AFRIpads can be reused for 12 months. Girls in the puberty education conditions received an education session (in October 2012) lasting ∼1.25 hours.^23^ Girls who transferred into the study schools during the trial were included in intervention delivery (if attending school at those times) alongside those in the trial and follow-up surveys as not to identify or stigmatise girls in the trial or discriminate against those transferring after the baseline from receiving resources. While girls transferring into the school could not be included in trial outcomes compared across conditions, their responses were incorporated into the final survey data set for secondary analyses. This maximised cross-sectional sample size in the survey data set.

The present study employs the final survey data from the trial to provide the first estimate of MHM consistent with the concept definition. This was used to estimate the association between poor MHM and health, education and psychosocial outcomes, alongside an assessment of the relative contribution of aspects of MHM to these experiences. MHM prevalence was assessed separately for those using the AFRIpads provided in the intervention and girls using other existing methods. The present study uses data from the final follow-up survey of the trial but does not compare across trial conditions, the work provides cross-sectional assessment of practices as reported in the survey and their associations with health, education and psychosocial well-being.

### Research questions

1. What are the self-reported MHM practices of girls in rural Uganda?
2. What is the prevalence of MHM consistent with the concept definition?
3. Is adequate MHM greater among those using reusable sanitary pads?
4. How do aspects of MHM, and pooled MHM, comparatively predict health, education and psychosocial well-being?

## Methods

This study was conducted and reported according to best practice guidelines in the Strengthening the Reporting of Observational studies in Epidemiology.^24^ Checklist for cross-sectional studies is reported in online supplementary materials 1.

10.1136/bmjopen-2016-012596.supp1 supplementary materials 1

### Participants

In total, 435 schoolgirls across the eight study schools completed follow-up surveys. This included girls who had been involved in the trial, and girls in the same classes who had joined the school after the trial started. All girls were surveyed to avoid selectively identifying those menstruating or involved in the trial, potentially stigmatising these girls. Two hundred five girls who reported in the survey that they had reached menarche were asked questions about menstrual management, and are included in this study. There were no a priori power analyses for this cross-sectional assessment as the study seeks to demonstrate the calculation of a prevalence estimate of MHM, and there was no past evidence from which to draw effect size estimates between aspects of MHM and health, education and psychosocial well-being outcomes assessed.

### Survey design

The survey was administered in November 2014. Trained local research assistants from the partner NGO (Plan International) used an English version of the survey loaded on iPads in the app *SurveyGizmo*. iPads were used in the field offline and data were uploaded to the online service *SurveyGizmo* at the end of each day once an internet connection could be established. Research assistants, all young women, verbally administered surveys in Lusoga (the local language) and inputted answers in English into the instrument. Girls were surveyed individually in a private place on the school grounds, for menstruating girls this lasted ∼30–40 min.

Survey items were designed following a pilot trial and qualitative research in Ghana,^3^ ^11^ where a number of similar items were also used. Additional questions were developed following feasibility and acceptability work leading to the selection of AFRIpads for the intervention study,^25^ and in consultation with stakeholders and partner NGO staff. To support best practice and transparency in reporting, a full list of survey items used is reported in online supplementary materials 2.

10.1136/bmjopen-2016-012596.supp2 supplementary materials 2

### MHM measure

Survey items capturing MHM practices were used to generate the measure of MHM prevalence.

*MHM practices:* Girls self-reported MHM practices through structured questions. To assess absorbent use, girls were asked: “What do you usually use to catch/absorb your menstrual period (MP)?”, and their free responses were recorded by research assistants as AFRIpad, cloth, toilet roll, sanitary pad or other. Few girls reported use of toilet roll so this was recoded to other, along with use of underwear alone and using mattress or sponge. In a follow-up question, girls were asked where they (or the person who gives them the absorbent) obtain it. This item was used to determine if girls were using old or new cloth. Girls were also asked: “How frequently do you change your sanitary protection?” with guidance from research assistants that per day meant in 24 hours.

For girls who reported using AFRIpads or cloths, follow-up questions captured washing and drying procedures. Girls responded yes/no to items: “Did you ever wash the AFRIpad/cloth?”, and to the follow-up: “Did you use soap?”. To assess drying, girls were asked: “Where did you hang the cloth to dry?” with responses including under the bed, outdoors, in the school dorm rooms and in a secret place. Free-text responses to the question included: in the bathroom, behind the toilet, in the grass and behind the house. Responses were back-coded into three categories: outside, hung inside or hidden inside. Girls were then asked: “How often did you wear the cloth/AFRIpad damp?” with response options *never*, *sometimes* and *usually*.

Girls were asked: “What do you usually do with your used sanitary protection when you have to change at home/school?” with response options *throw in latrine*, *throw in bush, dispose at community rubbish heap, bury* and *burn*. When asked about disposal at school, listed responses also included *go home to change. Wash and reuse* was a listed option for home disposal. For both questions, girls volunteered information and research assistants selected the appropriate category.

Those using reusable absorbents were asked: “Do you worry about being observed when washing the cloth/AFRIpad?” with yes/no response options.

*MHM criteria:* Self-reported MHM behaviours were used to generate a pooled, aggregate estimate of MHM consistent with the concept definition discussed in the background. Criteria for available aspects of MHM are detailed below, and the pooled estimate included absorbent cleanliness, adequate change frequency, hygienic washing and drying, and privacy. Criteria for each step were derived from background literature, although existing evidence is sparse. Each criterion was added sequentially and the number of girls considered to have adequate or inadequate MHM at each step reported in results. Absorbents were considered clean if they were AFRIpads, new cloth or sanitary pads, with old cloth and other items such as toilet paper, mattress, sponge or underwear alone considered inadequate. Changing absorbents three or more times per 24 hours was required to be considered adequate MHM, consistent with recent work^6^ and guidance.^1^ For reusable absorbents, MHM was considered inadequate if not washed with soap. Absorbents were considered to be hygienically dried if dried outside, rather than hung up or hidden inside. Never wearing absorbents damp was also required for adequate MHM. Finally, girls needed to report they were not worried about privacy for washing their absorbents. There were no appropriate items in the survey capturing washing the body or genitals, or items on the ability to change absorbents in privacy, which would have improved the MHM estimate. While disposal methods were reported, there was no consistent guidance on what could be considered an adequate method, so this item was not included in the overall estimate. These aspects were considered the *minimal* requirements for MHM, using best available evidence.

As criteria for MHM could be debated given the lack of evidence, an additional measure of MHM was used for comparison. For this pooled total more *relaxed* criteria were used. Both new and old cloth were considered to be adequate absorbents at step one, with the concession that if washed appropriately old cloth could be considered an adequate absorbent. Girls were only required to change their sanitary protection twice per day, although were still required to wash absorbents with soap and water. To meet criteria as having adequate MHM in the relaxed model, girls needed to dry absorbents outside or hung up inside; only those who dried their absorbent hidden away were considered to have poor MHM. The criteria that girls never wear absorbents damp remained unchanged, as did the requirement they felt they had adequate privacy to wash reusable absorbents. Girls were required to meet all relaxed criteria to be considered to have adequate MHM in this model.

### Survey measures

Additional survey measures assessed participant characteristics and hypothesised consequences of poor MHM.

*Participant characteristics:* Girls self-reported their age, grade in school, and how long it took them to walk to school. Data were also collected concerning access to water and soap at home and schools as yes/no responses to items asking “Do you have regular access to (water/soap) at (school/home)?”

*Health:* Girls were asked to report if during their *last* period they experienced: skin irritation or rashes in the pelvic area with a yes/no response. They were also asked if since the beginning of the school year they had experienced ‘any itching or burning in the pelvic area’, or ‘any white or grey discharge from their [your] vagina’ and could report if this was while on their period, at only other times, or both during their period and at other times. All girls reported that this was experienced either only during their period, or during their period and at other times, so only a dichotomous *experienced* response was used. Girls were asked: “Do you worry that other people can smell your menstrual period?” with yes/no responses.

*Education:* Girls reported if they felt their menstruation ever caused them to miss school or not to do their homework as part of a longer list of activities (including other items such as participating in sports or being around boys). Girls were asked: “Do you avoid standing in class to answer questions while on your menstrual period?” and “Do you find it difficult to concentrate at school when you have your menstrual period?” with yes/no options. The latter was followed with a multiple response item capturing the reasons for difficulties concentrating, listed in results tables.

*Psychosocial well-being:* Girls reported if during their *last* period they experienced embarrassment. They also reported if during their menstruation they felt ashamed or insecure or if this was the same as when they were not menstruating. Psychosocial well-being was assessed using the total score from the Strengths and Difficulties Questionnaire (SDQ^26^). The SDQ consists of 25, 3-point Likert scale items from 0 ‘not true’ to 2 ‘true’, with a midpoint of 1 ‘somewhat true’. The total score (0–40) uses summed scores from the four problem subscales of hyperactivity (eg, ‘I am easily distracted’), conduct problems (eg, ‘I usually do as I am told’), peer problems (eg, ‘I would rather be alone than with people of my age’) and emotional problems (eg, ‘I have many fears. I am easily scared’). The total difficulties score can range from 0–40. For 4–17-year-olds, current 4-band categorisation based on a UK survey population are average (0–14), slightly raised (15–17), high (18–19), very high (20–40). The questionnaire has been well validated and was a secondary outcome in the *Menstruation and the Cycle of Poverty* trial,^23^ with norms for different countries available online (http://www.sdqinfo.com).

### School attendance

Attendance data were recorded for girls participating in the *Menstruation and the Cycle ofPoverty* trial from baseline. For the present study, only follow-up attendance data were used. Research assistants from the partner NGO collected attendance for the fourth week of each of the three terms in 2014. This was collapsed to create a continuous variable: the per cent attendance out of the 15 days recorded. Week 4 of the school term was selected to avoid very low attendance periods coinciding with school fee collection at the start of term or agricultural practices, based on advice from site visits and local agents. Girls were not asked to record menstrual cycles, so attendance includes menstruating and non-menstruating days. Attendance was linked to survey responses through participant ID.

### Analyses

Analyses were conducted using Stata 14.0 (Stata Statistical Software: Release 14. College Station, Texas, USA: StataCorp LP. (program), 2015). Participant characteristics were detailed using descriptive statistics, as were the prevalence of MHM practices and consequences. Combined measures of MHM were reported using descriptive statistics, including 95% CIs of the total proportions of those with adequate and inadequate MHM. The χ^2^ statistic was used to compare the pooled MHM between those using AFRIpads and using other existing methods. Bivariate correlations were used to test the overlap of MHM aspects prior to use in multivariable models.

Univariate logistic regressions assessed the relationships between MHM practices and health, education and psychosocial outcomes. Washing absorbents (with or without soap) was not used as a predictor in these comparisons as almost all girls reported doing so. All of the girls who reported never wearing their absorbent damp had also dried their absorbent outside, so only the location of drying was used as a predictor in the models. All four aspects of MHM were included in multivariable logistic regressions to assess the individual contribution of each to hypothesised consequences. Univariate logistic regressions assessed the relationship between the combined measures of MHM and outcomes. For continuous outcomes of school attendance and total SDQ score, independent samples t-tests were used.

### Ethics

To participate in the trial, girls and their parent/caregiver provided written consent. Schools provided consent for participation throughout the duration of the study. At the start of the survey, girls provided verbal consent to participate. No girls declined participation. They were informed that they were free not to answer any question in the survey.

## Results

### Participant characteristics

A total of 205 menstruating girls were included in this study. Of them, 145 (70.7%) had been attending the schools in the trial at baseline. Of the 145 trial girls, 96 (66.2%) had received one of the tested interventions (puberty education alone: 36 (24.8%), pads alone: 40 (27.6%), and education and pads: 27 (18.6%). Of the 60 girls who had not been in the trial schools at baseline, 20 (33.3%) had received pads alone and one had received the education intervention. Interventions were identical for all girls, including transfers. Transfer girls received the interventions alongside girls in the trial from baseline to ensure there was no stigmatisation of either group. Eighty-one girls in the full sample had received no intervention, this included girls in the control condition, as well as girls who failed to receive their assigned intervention. Girls in this study were compared according to their self-reported menstrual hygiene practices.

Girls ranged from a self-reported age of 10–19 years (mean =14.20, SD=1.12). The average age at menarche was 12.82 years (SD=1.28). Table 1 describes the participant characteristics for this study.

**Table 1:** Participant characteristics (n=205)

	Per cent	N
Grade		
P3	0.5	1
P4	8.3	17
P5	21.5	44
P6	44.9	92
P7	24.9	51
Age		
10	0.5	1
11	0.5	1
12	1.0	2
13	21.5	44
14	44.9	92
15	20.5	42
16	8.8	18
17	1.0	2
18	1.0	2
19	0.5	1
Did you go to the same school last year?		
Yes	94.6	194
No	5.4	11
How long does it take you to get to school?		
10 min or less	19.8	33
11–30 min	37.7	63
31–60 min	37.7	63
>1 hour	4.8	8
* No answer*		*38*
Do you have regular access to:		
Soap at home? (n=200)	94.0	188
Water at home? (n=200)	99.0	198
Soap at school? (n=199)	27.1	54
Water at school? (n=199)	44.2	88

### MHM practices

Table 2 describes the girls’ MHM practices. Four did not report the type of absorbent used; therefore, were not asked follow-up questions. Thus the table reports on 201 girls, unless otherwise indicated. Almost 36% of the sample were using AFRIpads received as part of the intervention study as their primary absorbent. Of girls not using AFRIpads, most used cloth as absorbent. Of those using reusable absorbents, almost all reported washing them and using soap for every wash. Half of those who washed absorbents hid them to dry, most commonly under the bed. 23% reported wearing absorbents wet at least once. No girls reported disposing at community rubbish heaps, burning or burying pads.

**Table 2:** Self-reported menstrual hygiene management practices (n=201)

	Per cent	N
**Absorbent**		
What do you usually use as menstrual absorbent?		
AFRIpad	35.8	72
New cloth	14.9	30
Old cloth	30.9	62
Sanitary pad	9.0	18
Other (incl. toilet paper, underwear, mattress, sponge)	9.5	19
How frequently do you change your sanitary protection?		
1× per day	6.1	12
2× per day	33.2	65
3× per day	49.0	96
4× per day	9.7	19
5+ times per day	2.0	4
* Missing*		*5*
**Washing reusable absorbents**		
Did you wash the absorbent? (n=158)*		
Yes	95.7	154
No	1.9	3
* No answer/don't know*	2.5	4
Did you use soap? (n=154)†		
Never	0	0
Sometimes	1.9	3
Every time	98.1	151
**Drying reusable absorbents**		
Where did you dry the absorbent? (n=154)†		
Hidden inside (eg, under bed)	47.4	73
Hung up inside (eg, girls dorm, bathroom)	11.0	17
Outside	41.6	64
How often did you wear the absorbent damp? (n=154)†		
Never	77.3	119
Sometimes	6.5	10
Usually	16.2	25
**Absorbent disposal**		
What do you usually do with your used sanitary protection when you have to change at school? (n=158)‡		
Throw in latrine	12.3	19
Take home to wash or dispose	85.7	132
* Other*	*12.0*	*3*
What do you usually do with your used sanitary protection when you change at home? (n=199)		
Throw in latrine	11.6	23
Put in trash	0.5	1
Wash and reuse	87.9	175
**Privacy**		
Do you worry about being observed when washing the absorbent? (n=154)†		
Yes	73.4	113
No	26.6	41

*Of girls using reusable menstrual absorbents (AFRIpads, cloth).

†Of girls using reusable menstrual absorbents (AFRIpads, cloth) who reported washing their absorbent.

‡Only asked of girls who reported they ‘couldn't go whole day at school without changing absorbent’.

### Prevalence estimate: MHM

By adding each available aspect of MHM assessed, the overall prevalence of MHM was estimated. Table 3 displays the proportion of girls who met criteria at each step. That is, the proportion of girls remaining after the introduction of that requirement for MHM. Each additional aspect of MHM resulted in fewer girls qualifying. There were particularly large drops following the introduction of changing frequency, drying and privacy. After the inclusion of available aspects, only 9.5% of the sample qualified as having adequate MHM using minimal criteria. The prevalence of inadequate MHM did not differ between those who used AFRIpads (88.6%) and those using existing methods (90.5%; χ^2^ (1)=0.12, p=0.729).

**Table 3:** Estimated prevalence of menstrual hygiene management using minimal and relaxed criteria

	MHM criteria (minimal)		MHM criteria (relaxed)	
	AFRIpad users N=72	Usual practice N=129	AFRIpad users N=72	Usual practice N=129
	Application of criteria % (n) retained		Application of criteria % (n) retained	
Clean absorbent	(Criteria: AFRIpad, new cloth, or sanitary pad)		(Criteria: AFRIpad, old or new cloth, sanitary pad)	
	100 (72)	37.2 (48)	100 (72)	85.3 (110)
Changed frequently	(Criteria: 3 times or more)		(Criteria: 2 times or more)	
	66.7 (48)	19.4 (25)	93.1 (67)	80.6 (104)
Washed with soap*	(Criteria: washed absorbent with soap)		(Criteria: washed absorbent with soap)	
	65.3 (47)	18.6 (24)	90.3 (65)	79.1 (102)
Dried adequately*	(Criteria: absorbent dried outside)		(Criteria: absorbent dried outside or hung inside)	
	29.2 (21)	10.1 (13)	56.9 (41)	45.7 (59)
	(Criteria: absorbent never worn damp)		(Criteria: absorbent never worn damp)	
	29.2 (21)	10.1 (13)	56.9 (41)	41.1 (53)
Privacy for washing*	(Criteria: not worried about being observed washing absorbent)		(Criteria: not worried about being observed washing absorbent)	
	11.1 (8)	8.5 (11)	19.4 (14)	23.3 (30)
	% (95% CI) (n)	% (95% CI) (n)	% (95% CI) (n)	% (95% CI) (n)
Meet available criteria	11.1 (5.6% to 21.0%) (8)	8.5 (4.7% to 14.8%) (11)	19.4 (11.7% to 30.5%) (14)	23.3 (16.7% to 31.4%) (30)
Prevalence of poor MHM	88.9 (79.0% to 94.4%) (64)	91.5 (85.2% to 95.3%) (118)	80.6 (69.5% to 88.3%) (58)	76.7 (68.6% to 83.3%) (99)
	Total % (95% CI) (n)		Total % (95% CI) (n)	
Met available criteria	9.5 (6.1% to 14.4%) (19)		21.9 (16.7% to %28.2) (44)	
Prevalence of poor MHM	90.5 (85.6% to 93.9%) (182)		78.1 (71.8% to 83.3%) (157)	

*If reusable absorbent (AFRIpad, new/old cloth).

MHM, menstrual hygiene management.

As noted previously, there is a lack of evidence for MHM criteria. Thus, more relaxed criteria were applied for comparison (table 3, right columns). Despite these changes, the overall prevalence of poor MHM remained high and there was no significant difference between those using AFRIpads (80.6%) and those using existing methods (76.7%; χ^2^ (1)=0.20, p=0.655).

While the study aimed to use each aspect of MHM to predict outcomes, almost all girls reported washing absorbents with soap. Thus, groups were not large enough for reliable comparison in χ^2^ or logistic regression so this criterion was dropped from analysis of association with outcomes. As noted in the analysis section, drying criteria were collapsed. The minimal MHM criteria from table 3 (ie, new cloth, AFRIpad or sanitary pads considered clean absorbent, and outside alone considered adequate drying) were used for comparison with health, education and psychosocial outcomes.

To check for multicollinearity and associations between the different aspects of MHM, bivariate correlations were compared between the clean absorbents (AFRIpad, new cloth, sanitary pad), frequency of absorbent change (3+ times), drying adequately (outside) and having adequate privacy. There was only one significant correlation between girls feeling they had adequate privacy to wash absorbents and drying absorbents adequately (outside), r=0.42, p<0.001. All other associations were very low (r<0.08).

### Consequences of poor MHM

Table 4 presents the proportion of girls reporting negative health, education, and psychosocial outcomes. Approximately half of the sample reported discomfort, possibly indicating health consequences. Less than 20% of girls stated that menstruation caused them to miss school, although over half reported not standing in class to answer questions, and finding it difficult to concentrate when menstruating. Discomfort, fear of soiling and menstrual pain were the most common reasons for difficulty concentrating. Many girls reported embarrassment, shame and insecurity associated with menstruation.

**Table 4:** Prevalence of proposed consequences of poor MHM (n=201)

	Per cent	N
**Health/comfort**		
Skin irritation/rashes in pelvic area during last MP (n=153)*	54.3	83
Itching or burning in the pelvic area (since start of the school year) (n=199)	60.3	120
White or green discharge (since start of the school year) (n=199)	47.2	94
Do you worry people can smell your MP? (yes)	70.2	141
**Education**		
Does your MP ever cause you to: (n=185)*		
Miss school?	18.4	34
Not do your homework?	7.6	14
School attendance at intervention follow-up (n=144) M (SD)	81.06	(18.58)
Do you avoid standing in class to answer questions on your MP? (n=198)	64.7	128
Do you find it difficult to concentrate at school when you have your MP? (n=198)	51.0	101
Reasons it is difficult to concentrate during MP (n=101)		
Actual soiling	24.8	25
Fear of soiling	72.3	73
Scent	15.8	16
Discomfort	49.5	50
Actual teasing	4.0	4
Fear of teasing	21.8	22
Cramps	54.5	55
**Psychosocial well-being**		
Strengths and Difficulties Questionnaire (SDQ) total score M (SD)	19.11	(5.31)
Did you experience embarrassment during your last MP? (n=153)*	45.8	70
During your MP do you feel ashamed?	69.2	139
During your MP do you feel insecure?	69.2	139

*n is lower than 201 resulted in part from iPad issues where, in about 40 cases, the appropriate page froze or failed to load, additional missing resulted from girls not providing an answer to the question.

MHM, menstrual hygiene management; MP, menstrual period.

### Relationships between MHM aspects and proposed consequences

Table 5 displays the univariate and multivariable relationships between MHM aspects, pooled MHM estimates (minimal and relaxed), and health, education and psychosocial well-being.

**Table 5:** Univariate and multivariable comparisons of aspects of MHM, combined measures of MHM and hypothesised consequences

	Absorbent type		Absorbent change		Absorbent drying		Privacy (for washing)		MHM (minimal)		MHM (relaxed)	
	Clean	Unclean	3+ times	<3 times	Outside	Inside/hid	No concern	Concern	Adequate	Inadequate	Adequate	Inadequate
	Per cent (N)	Per cent (N)	Per cent (N)	Per cent (N)	Per cent (N)	Per cent (N)	Per cent (N)	Per cent (N)	Per cent (N)	Per cent (N)	Per cent (N)	Per cent (N)
		OR (95% CI)		OR (95% CI)		OR (95% CI)		OR (95% CI)		OR (95% CI)		OR (95% CI)
**Health/comfort**												
Itching or burning in the pelvic area (since start of the school year)	59.7 (71)	61.3 (49)	60.2 (71)	60.5 (49)	58.6 (58)	62.0 (62)	54.4 (43)	64.2 (77)	52.6 (10)	61.1 (110)	54.6 (24)	61.9 (96)
		1.07 (0.60 to 1.91)		1.01 (0.57 to 1.81)		1.15 (0.65 to 2.04)		1.50 (0.84 to 2.67)		1.41 (0.55 to 3.65)		1.36 (0.69 to 2.67)
OR_adj_ (95% CI)		1.10 (0.61 to 1.98)		0.98 (0.54 to 1.76)		0.96 (0.51 to 1.82)		1.54 (0.80 to 2.93)				
White or green discharge (since start of the school year)	44.5 (53)	51.3 (41)	48.3 (57)	45.7 (37)	53.5 (53)	41.0 (41)	44.3 (35)	49.2 (59)	31.6 (6)	48.9 (88)	47.7 (21)	47.1 (73)
		1.31 (0.74 to 2.31)		0.90 (0.51 to 1.59)		0.60 (0.34 to 1.06)		1.22 (0.69 to 2.15)		2.07 (0.75 to 5.69)		0.98 (0.50 to 1.91)
OR_adj_ (95% CI)		1.36 (0.76 to 2.43)		0.80 (0.45 to 1.44)		0.47* (0.24 to 0.88)		1.78 (0.92 to 3.43)				
Worry people can smell your MP?	65.8 (79)	76.5 (62)	68.9 (82)	73.0 (59)	74.3 (75)	66.0 (66)	58.2 (46)	77.9 (95)	63.2 (12)	70.9 (129)	59.1 (26)	73.3 (115)
		**1.69**† **(0.90 to 3.20)**		1.16 (0.62 to 2.15)		0.68 (0.37 to 1.24)		2.52** (1.36 to 4.69)		1.42 (0.53 to 3.80)		**1.90**† **(0.94 to 3.81)**
OR_adj_ (95% CI)		**1.88**† **(0.96 to 3.68)**		0.94 (0.49 to 1.81)		0.34** (0.16 to 0.73)		4.51*** (2.09 to 9.77)				
Education												
School attendance M (SD) (n=144)	82.10 (18.33)	79.39 (19.04)	**84.89 (14.28)**	76.40** (22.23)	79.00 (21.54)	83.19 (14.82)	80.12 (21.14)	81.67 (16.86)	86.19 (13.95)	80.51 (18.98)	81.96 (20.07)	80.79 (18.19)
Do you avoid standing in class to answer questions on your MP?	59.3 (70)	72.5 (58)	63.6 (75)	66.3 (53)	59.6 (59)	69.7 (69)	53.2 (42)	72.3 (86)	26.3 (5)	68.7 (123)	45.5 (20)	70.1 (108)
		1.81 (0.98 to 3.34)		1.13 (0.62 to 2.04)		1.56 (0.87 to 2.81)		2.30** (1.26 to 4.17)		6.15** (2.11 to 17.91)		2.82** (1.42 to 5.60)
OR_adj_ (95% CI)		1.97* (1.05 to 3.71)		1.05 (0.56 to 1.95)		1.14 (0.58 to 2.22)		2.31* (1.18 to 4.53)				
Do you find it difficult to concentrate at school when you have your MP? (n=198)	46.6 (55)	57.5 (46)	49.2 (58)	53.8 (43)	52.5 (52)	49.5 (49)	39.2 (31)	58.8 (70)	42.1 (8)	52.0 (93)	31.8 (14)	56.5 (87)
		1.55 (0.87 to 2.75)		1.20 (0.68 to 2.12)		0.89 (0.51 to 1.55)		2.21** (1.24 to 3.95)		1.49 (0.57 to 3.87)		2.78** (1.37 to 5.66)
OR_adj_ (95% CI)		**1.69**† **(0.93 to 3.05)**		1.05 (0.58 to 1.91)		0.55 (0.29 to 1.07)		3.03** (1.54 to 5.99)				
Psychosocial well-being												
Strengths and Difficulties Questionnaire M (SD)	18.65 (5.24)	19.80 (5.37)	18.75 (5.42)	19.65 (5.13)	18.91 (5.34)	19.32 (5.29)	18.84 (5.42)	19.30 (5.25)	17.11 (6.30)	19.32 (5.17)	18.25 (5.80)	19.36 (5.16)
Did you experience embarrassment during your last MP (n=153)	47.8 (43)	42.9 (27)	46.2 (42)	45.2 (28)	48.8 (39)	42.5 (31)	54.4 (31)	40.6 (39)	50.0 (6)	45.4 (64)	38.7 (12)	47.5 (58)
		0.82 (0.43 to 1.57)		0.96 (0.50 to 1.84)		0.78 (0.41 to 1.47)		0.57 (0.30 to 1.11)		0.83 (0.26 to 2.70)		1.43 (0.64 to 3.21)
OR_adj_ (95% CI)		0.76 (0.39 to 1.47)		1.02 (0.52 to 1.98)		0.93 (0.46 to 1.86)		0.57 (0.28 to 1.17)				
During your MP do you feel ashamed? (n=201)	66.7 (80)	72.8 (59)	69.8 (83)	68.3 (56)	72.3 (73)	66.0 (66)	59.5 (47)	75.4 (92)	52.6 (10)	70.9 (129)	56.8 (25)	72.6 (114)
		1.34 (0.72 to 2.49)		0.93 (0.51 to 1.72)		0.74 (0.41 to 1.36)		2.09* (1.13 to 3.84)		2.19 (0.84 to 5.70)		2.01* (1.01 to 4.03)
OR_adj_ (95% CI)		1.44 (0.76 to 2.73)		0.79 (0.42 to 1.49)		0.44* (0.21 to 0.91)		3.18** (1.54 to 6.57)				
During your MP do you feel insecure?	68.3 (82)	60.4 (57)	69.8 (83)	68.3 (56)	68.3 (69)	70.0 (70)	60.8 (48)	74.6 (91)	63.2 (12)	69.8 (127)	61.4 (27)	71.3 (112)
		1.10 (0.60 to 2.03)		0.93 (0.51 to 1.72)		1.08 (0.59 to 1.97)		1.90* (1.03 to 3.48)		1.35 (0.50 to 3.60)		1.57 (0.78 to 3.15)
OR_adj_ (95% CI)		1.16 (0.62 to 2.16)		0.86 (0.46 to 1.60)		0.78 (0.39 to 1.54)		2.16* (1.09 to 4.28)				

Adequate absorbent OR, 1.00; changed frequently OR, 1.00; drying outside OR, 1.00; don't worry about privacy OR, 1.00; adequate MHM strict OR, 1.00; adequate MHM relaxed OR, 1.00.

OR_adj_, OR for multivariable models where all aspects of MHM were included in the model.

*p<0.05, **p<0.01, ***p<0.001, †p<0.10.

MHM, menstrual hygiene management; MP, menstrual period.

There were no significant associations with itching or burning, despite a consistently higher rate of report among those with poorer MHM. Drying outside had the reverse relationship to discharge than expected, with less white or green discharge reported among those drying inside or hidden. This unexpected direction of effect was also found for girls’ fears that others could smell their menses. Unclean absorbents and inadequate privacy were associated with increased concerns about odour in multivariable comparison.

Only one aspect of MHM was associated with school attendance, with those changing three times a day or more having a higher attendance rate. Unclean absorbents and privacy were associated with higher odds of avoiding standing in class to answer questions, as were pooled MHM estimates. Only adequate privacy for washing absorbents was associated with difficulties concentrating, although having a clean menstrual absorbent trended towards significance with an almost 10% difference in reports. Drying practices were associated with shame in the opposite direction to that expected. Adequate privacy for washing was associated with shame and insecurity.

Despite very large percentage differences in the reports of consequences between those categorised as having adequate and inadequate MHM, the small number of girls with adequate MHM meant there was insufficient power to demonstrate statistically significant effects. Per cent differences should be noted, however, with the potential for large effect sizes to be identified in larger samples.

## Discussion

### Prevalence of poor MHM

This study was the first to provide a prevalence estimate for MHM consistent with the concept definition, ‘women and adolescent girls using a clean menstrual management material to absorb or collect blood that can be changed in privacy as often as necessary for the duration of the menstruation period, using soap and water for washing the body as required, and having access to facilities to dispose of used menstrual management materials’.^16^ Of the total sample, 90.5% (95% CI 85.6% to 93.9%) had inadequate MHM. This did not differ between those using reusable pads and those using other existing methods (71.3% cloth, 14.0% disposable sanitary pads, 14.7% other methods including toilet paper and underwear alone). Even when more relaxed criteria were used (use of sanitary pad, AFRIpad, old or new cloth, that was changed two or more times per day, with absorbents washed with soap and dried hung outside or inside, absorbents never worn damp, and girls reporting they were not worried about being observed washing their absorbent), the prevalence of poor MHM was 78.1% (95% CI 71.8% to 83.3%), and did not differ for those using reusable pads. By employing the current definition of the concept, this work has shown that reporting individual aspects of MHM alone underestimates the extent of deprivation. Furthermore, when MHM is considered as a whole, girls using reusable sanitary pads provided as part of the intervention trial did not report a lower prevalence of poor MHM. As such, the use of reusable sanitary pads alone may not improve girls’ menstrual hygiene.

MHM behaviours reported by the sample are consistent with the rural context and poverty in the study area. The sample may be more disadvantaged, at least in terms of menstrual hygiene, than some past studies. A higher proportion of girls (who did not use AFRIpads) reported using cloth compared with studies of girls in rural India (pooled prevalence: 63%^12^), and single-study estimates from East Africa (24% in rural Kenya;^18^ 56% in Ethiopia^9^). A similarly high rate of cloth use (87%) was recently reported in the Rukungiri district of Uganda.^27^ There are far fewer studies with which to compare reports of washing or drying practices. Past studies have also failed to adequately report questions used to capture MHM practices, which limits comparison (J Hennegan. Menstrual hygiene management and human rights: the case for an evidence-based approach. Under review). This study focused on the absorbent girls reported using *most frequently*.

Facilities for MHM were comparable across all study schools. Latrines were gender-separated but had few adequate doors or locks, and no access to water. Girls reported going home to change absorbents, and all stated that they washed and dried reusable absorbents at home, rather than at school. No survey items asked about transporting menstrual absorbents home for cleaning, which may have presented an additional challenge for girls and resulted in anxiety or stigma. Improvements to facilities for changing and cleaning absorbents at the schools may have improved MHM. While advocates have increasingly focused on the provision of facilities at school for MHM,^22^ this was not the focus of the present study and the washing and drying practices reported in this sample were undertaken at home, rather than at school.

### Consequences of poor MHM

High rates of negative outcomes were observed. Many girls reported genital irritation, discharge and concerns about odour. While self-reported symptoms may not be the best predictor of laboratory-confirmed infections,^8^ girls’ discomfort and symptoms which may cause distress, represent important health outcomes. Over half of the sample reported not standing to answer questions and difficulties concentrating in school. Sixty-nine per cent of girls reported shame and insecurity during menstruation. High SDQ scores in the sample have been discussed elsewhere,^2^ in part consistent with high scores reported in low-income contexts, and a possible bias towards affirmative responses.^23^ Large per cent differences were observed using the pooled MHM measures for all consequences assessed. Unfortunately, the small proportion of girls with adequate hygiene meant there was insufficient power to detect many effects. Nevertheless, findings support the value of considering all aspects of MHM together.

There were few associations between aspects of MHM and health symptoms. As noted above this may reflect the poor validity of girls’ self-reports.^8^ Concerns about odour were associated with absorbent type, drying practices and privacy. AFRIpads provided as part of the *Menstruation and the Cycle of Poverty* trial were ∼7–8-months-old at the time of follow-up survey. As AFRIpads last 12 months, the deterioration of the pads is unlikely to have contributed to outcomes. However, if poorly maintained, the pads may not have been a clean absorbent, or associated with reduced irritation and infection.

Using unclean absorbents was associated with concerns about odour, difficulties in school concentration and standing to answer questions. Actual soiling or fear of soiling and odour may mediate the identified relationship with school participation and should be investigated in future work. Drying absorbents adequately was associated in an unanticipated direction with odour concerns and reports of discharge. There may be multiple explanations for this finding. First, girls drying absorbents outside may place them on unclean surfaces or on the ground, which may increase contamination. Drying outside may provoke concerns that absorbents will be seen, thus they may not be left to dry adequately. Consistency of reports between drying outside and never wearing absorbents damp, however, works against this interpretation. While replication would be needed, findings have interesting implications for interventions which encourage outside drying. If conditions outside are unclean, this may cause unintended harm.

Concerns about privacy for washing absorbents were associated with many consequences including shame, insecurity and disengagement at school. The privacy item captured worry about privacy for washing absorbents, so associations may have been driven by girls’ trait anxiety. However, it would be difficult to measure privacy more objectively, as adequacy is in many ways subjective.^7^ Girls’ feelings of safety are likely to reflect some individual differences. In interpreting the associations between privacy for washing and outcomes, one explanation may be that girls who felt they were likely to be observed did not wash absorbents as well as those without this pressure. As noted above, high rates of reported washing with soap ‘always’ are likely to be misleading. This also did not capture the quality of washing. Those washing absorbents quickly may have been unable to get them clean and properly prepared for future use and minimal odour. More research is needed to investigate the validity and reliability of these measures, and provide recommendations on criteria for adequate washing for health and odour prevention. Reports of perceived privacy for changing and drying absorbents represent important parts of the MHM concept definition which were not able to be captured by available measures in this study.

Only the frequency of absorbent change was significantly associated with school attendance, although the sample size was limited. Consistent with guidelines for MHM, girls changing their absorbents three or more times per day had a greater percentage of school days attended. This may be due to a lower rate of soiling, or concerns about soiling, among these girls as they used a fresh absorbent more often. This group of girls may feel more confident to change their absorbents at school, leading to greater attendance. Past literature^28^ and reports from local agents in the field in this study suggest many girls go home to change their absorbents. Interventions proposing to improve the quality of facilities for changing at school may therefore be effective in improving attendance for more girls. It should be noted that in order for girls to change three or more times per day they must have access to sufficient absorbents (cloth or pads) to do so, thus this may represent a more advantaged group and this study was unable to adjust for such sociodemographic confounds.

### Strengths and limitations

The present study was undertaken in a difficult-to-reach population of adolescent girls in rural Uganda. The use of local research assistants and language improved access to the population and facilitated comfort with the interviewers. The prevalences of MHM behaviours are heavily dependent on the reliability of girls’ reports and questions used, an issue that pervades MHM studies. The study found almost universal washing with soap. While girls report that soap and water is available to them at home, qualitative interviews, site reports and reports from the field suggest always washing absorbents with soap to be unlikely. Social desirability is likely to have influenced self-reported behaviours, urogenital symptoms and the impact of menstruation on schooling. Interviewers were from the local NGO, well known in the area, with a recent campaign focused on girls’ education (https://plan-international.org/what-we-do/because-i-am-girl). In addition, many had been involved in delivering the AFRIpads to the girls. It is unclear if written surveys would have reduced social-desirability in responses, and may have introduced other biases given the low levels of literacy in the area. Additionally, this would have required costly translation of surveys and girls’ responses.

The use of an objective measure of school attendance meant this was not subject to girls’ self-reports, however, means these data reflect attendance across all days including menstruating and non-menstruating days. This means effect sizes are limited, and the impact of confounds on attendance which pervade across non-menstrual days may have a greater influence than if only menstruating school days were compared. Future studies of menstrual-specific absence would provide a more specific measurement of menstrual and MHM-related absenteeism, but may be difficult to obtain as they are reliant on receiving girls’ self-reports of their cycle. The distribution of AFRIpads and education in the sample mean that the prevalences of MHM behaviours and consequences reported may not reflect practices prior to the interventions. As noted previously, a proportion of girls (n=60) not included in the *Menstruation and the Cycle of Poverty* trial results were included in this study. While these girls were not included in the trial sample, many had been attending the study schools from very near the baseline assessment, and were comparable to girls in the trial, with many also receiving the interventions alongside the trial girls. This study used best available evidence to guide criteria for MHM estimates and predictive models; however, as noted, there is a dearth of literature to guide hygiene recommendations and research.

The cross-sectional and correlational nature of the research precludes causal inference. With regard to the prevalence of menstrual hygiene, this study compared girls who reported using AFRIpads provided in the trial as their primary absorbent with those using other methods when calculating MHM prevalence. While this presents a cross-sectional assessment of menstrual hygiene when using a reusable product, it does not reflect the effectiveness of providing reusable pads in an intervention. Girls were not compared across the intervention arms, rather across the primary absorbent used. Those in the reusable pad arms, who may have received AFRIpads but not used them, were grouped according to the absorbent they used most often. Cross-sectional relationships between MHM and health, education and psychosocial well-being outcomes are limited by the inability to adjust for sociodemographic confounds. It is likely that greater access to resources is associated with better MHM and proposed consequences. Girls with greater access to resources are likely to have better health outcomes, family support for school attendance, fewer other challenges to psychosocial well-being. Thus, the study would have been greatly improved by the ability to adjust for sociodemographic factors such as parental education and poverty.^12^ The small sample size further limited these analysis. As this work was exploratory, and the first to estimate a combined measure of MHM and assess multiple aspects of MHM and their relationship to outcomes, there was very limited literature on which to base a priori power analyses and none were undertaken. This study provides some indication of expected effect sizes to enable power analyses in future studies.

The nature of, and need for, a pooled estimate of MHM could be questioned. As argued in the background to this paper, this is useful for establishing the state of MHM in different populations and advocating for attention. It also promotes use of the MHM term consistent with its definition, and provides greater clarity around what should be considered predictors and consequences of MHM, rather than aspects of it. This highlights the need for alternate terminology capturing other factors such as menstrual taboos that impact on girls’ menstrual management but are not MHM. Until evidenced guidelines are developed, and comparable measures of MHM have been tested and used across studies, it is not advised to present only pooled estimates. The individual aspects that make up MHM will always be important individual factors, as indicated by the differential relationships with outcomes identified in this work. This paper presents a worked example of how the MHM concept could be operationalised. The aspects of the definition, placing equal value on each of these and the cut-off points used in this work could all be debated. More work is needed to guide research and practice.

Finally, the individual items available to appraise MHM in this study could be improved. MHM literature has paid insufficient attention to measurement issues and there are presently no validated questionnaires for assessing practices such as absorbent washing and drying. The measures in the present study could be improved on in future work. Questions often asked about how girls ‘usually’ dry or dispose of absorbents which may result in greater social desirability in responses than asking about the last menstrual period. Response options for drying items need improvement. Field work in the study found that girls drying absorbents outside would often do so under another piece of fabric which may reduce the UV benefits of drying outside. Drying pads ‘hidden’ lacks specificity which could be improved on in future work.

### Implications for research and practice

Recent calls for action on MHM state the need for a strong quantitative evidence base.^22^ This cannot be achieved without consistent reporting of MHM as defined. Prevalence estimates are needed to advocate effectively for action, establish hypothesised consequences and measure improvements in intervention trials. Slow movement to address MHM deficits to date may reflect the present absence of quantitative prevalence figures and links to consequences. Confusion between what represents a predictor or aspect of poor MHM will continue without guidance and tools for measuring MHM as defined. This delays the development of a detailed, and quantitatively supported, problem theory of MHM. There is an urgent need for guidelines detailing the MHM concept and criteria for adequacy in each aspect, as well as research guidance to establish a rigorous primary evidence base in this field.

Minimal correlations between aspects of MHM and differential associations between aspects and consequences in multivariable models demonstrate unique impact of each facet. Findings suggest all MHM aspects must be considered in testing links with hypothesised consequences. Null results, particularly for psychosocial consequences, suggest more predictors may need to be considered. Hygiene management is not the only menstruation-related challenge facing girls in low-income contexts. Fear around menstruation due to lack of understanding, taboos and stigma may contribute to these outcomes. While properly defining and measuring aspects of MHM, future studies must also investigate these other contributors to outcomes.

The present study suggests that using AFRIpads was not associated with a higher prevalence of adequate MHM. While this study does not compare intervention conditions, findings suggest that improving only one aspect of MHM, the absorbent used, may not improve MHM overall. The *Menstruation and the Cycle of Poverty* trial did find that across conditions the provision of AFRIpads was effective in improving school attendance.^23^ Thus, it may be that addressing only one aspect of MHM is sufficient to improve outcomes, as suggested by differential associations in this study, but is insufficient to provide girls with their full right to MHM.^7^ The provision of a reusable product may have a negligible impact on MHM due to their dependency on washing, drying and privacy facilities which are unaffected by product-provision interventions. In the present study, it may be that girls needed additional training in AFRIpad use beyond what was provided to improve cleaning practices. Providing disposable pads which do not require washing or drying, and may be quicker to change, might have a larger impact on MHM. This should be investigated in future work. Inserted products including menstrual cups and tampons were not considered culturally appropriate in this population, but may present another alternative to reusable pads for future studies.^25^ ^29–33^ In interpreting the primary trial results, which found improvements in school attendance following reusable pad provision,^23^ it is likely that improvements to unmeasured aspects of menstrual management such as improving teachers’ awareness of girls’ needs or improved social support by prompting girls to discuss menstruation may have contributed to these effects. More quantitative epidemiological studies and in-depth analysis of trial results, such as mediation analyses, are needed to fully understand the pathways of effect in interventions and maximise their future effectiveness.
